# Ovarian structures modulate cellular aggregation and gene expression in oviductal isthmus cells

**DOI:** 10.1590/1984-3143-AR2025-0075

**Published:** 2026-01-26

**Authors:** Isabelle Cristina Pereira, Ana Carolina Bahia Teixeira, Raphael Rocha Wenceslau, Erika Cristina Jorge, Paola Maria da Silva Rosa, Julia Meireles Nogueira, Jade Carceroni de Sousa Carvalho, Gabriela Ponath Peruzzo, José de Oliveira Carvalho, Leticia Zoccolaro Oliveira

**Affiliations:** 1 Departamento de Clínica e Cirurgia Veterinárias, Escola de Veterinária, Universidade Federal de Minas Gerais – UFMG, Belo Horizonte, MG, Brasil; 2 Departamento de Morfologia Instituto de Ciências Biológicas – ICB, Universidade Federal de Minas Gerais – UFMG, Belo Horizonte, MG, Brasil; 3 Departamento de Medicina Veterinária, Universidade Federal do Espírito Santo – UFES, Alegre, ES, Brasil

**Keywords:** isthmus cellular aggregates, oviductal explants, gene expression

## Abstract

The presence of follicles and the corpus luteum (CL) in the ovarian surface plays a key role in determining the morphological and molecular fate of the female reproductive tract. However, the specific response of the isthmus epithelium to these ovarian structures remains poorly understood. This study hypothesizes that distinct ovarian structures differentially affect both the cellular aggregate-forming capacity of oviductal isthmus cells and the expression of *ESR1, ESR2*, and *PGR* genes. Reproductive tracts were categorized into three groups: ovaries with ≤5 mm follicles (small follicles group; SF); ovaries with follicles between 8-10 mm (large follicles group; LF); and ovaries containing active corpus luteum (CL Group). Isthmus cells from the three groups were cultivated to form cellular aggregates (oviductal explants) during 24 h. Moreover, the expression levels of *ESR1, ESR2*, and *PGR* genes were analyzed in the isthmus cells of the experimental groups. The isthmus cells of LF group showed an increased number of cellular aggregates than SF and CL group. Additionally, the SF group presented more aggregates than the CL group. Gene expression analysis revealed that *ESR1* expression was higher in the SF group than in the LF group. Moreover, *PGR* expression was greater in the CL than in the SF group, as well as in the LF than in the SF group. In conclusion, ovarian structures impact the cellular aggregate formation capacity and the gene expression of ovarian steroid receptors in isthmus cells.

## Introduction

The oviduct is a tubular segment of the female reproductive tract, composed of three distinct regions: the infundibulum, ampulla, and isthmus (Hunter, 2012). Early studies described the oviducts as passive conduits, functioning exclusively in the transport of gametes and embryos through their luminal compartments. However, recent investigations have demonstrated that the oviducts function as dynamic structures, with their secretory activity and cellular composition undergoing cyclical modifications throughout the estrous cycle mainly mediated by sex steroids (Gonella-Diaza et al., 2018). Taken together, the distinct morphological and functional features of each region contribute to the establishment of an optimal environment for gamete transport and provide a temporary site for early embryonic development following fertilization.

In terms of oviductal histology, the epithelium is composed primarily of two main cell types: ciliated and secretory cells. The proportion of ciliated cells increases along the isthmus-to-infundibulum axis, whereas the ampulla contains the highest concentration of secretory cells (Donnez et al., 1985), which are responsible for the synthesis and secretion of the components that constitute the oviductal fluid (Binelli et al., 2018; Hunter, 2012). Over the course of the estrous cycle, oviductal tissue passes through morphological, molecular and functional modifications (Gonella-Diaza et al., 2017). From the follicular to the luteal phase, a gradual reduction in secretory cell populations occurs (Abe, 1994), presumably modulated by ovarian steroid hormones secreted by ovarian structures such as dominant follicles and the corpus luteum, which predominate at each respective stage.

Ovarian structures, such as follicles and the corpus luteum, secrete steroid hormones. Follicles secrete both estrogen (E2) and progesterone (P4), whereas the corpus luteum secretes progesterone, at varying concentrations throughout the different stages of the estrous cycle. The binding of these hormones to their respective receptors, *ESR1* (encoding the ERα receptor), *ESR2* (encoding the ERβ receptor), and *PGR* (encoding the progesterone nuclear receptor) modulates the transcription of region-specific genes within the bovine isthmus (Gonella-Diaza et al., 2015).

Comprehensive studies across species have been conducted to elucidate the role of these cell types during oviducts transport. As example, an in vitro cell culture model using cellular aggregates, secretions, and explants has been developed. This model has enabled investigations into the interactions between these components and sperm cells; their association with male fertility; the correlation between cellular aggregates and the viability of sexed and conventional spermatozoa; and their interactions with embryos and embryo-derived substances (Carvalho et al., 2018; Ferraz et al., 2017; Gualtieri and Talevi, 2003; Rajagopal et al., 2006; Ulbrich et al., 2010)

Despite these findings on the physiological influence of steroid hormones on the oviducts, little is known about how different types of ovarian structures and/or the different stages of the estrous cycle influence oviductal cells, particularly those capable of forming cellular aggregates. Therefore, the aim of this study was to assess the capacity of bovine oviductal isthmus cells, obtained from reproductive tracts exhibiting distinct ovarian characteristics, to form cellular aggregates, as well as to evaluate the expression of the *ESR1*, *ESR2*, and *PGR* genes in oviducts associated with predominant ovarian structures.

## Methods

This study followed research ethical guidelines and do not require a CEUA protocol since no live animals were included in the study.

### Experimental design

Experimental groups were established using reproductive tracts collected from slaughtered cows and categorized according to ovarian morphological characteristics into three groups, categorized by the presence of small follicles (SF group), large follicles (LF group), or corpus luteum (CL group) within the ovaries. To select the experimental groups based on ovarian follicle size, follicles were externally measured through the ovarian surface. Isthmus cells in the SF group were obtained from reproductive tracts in which the ovary ipsilateral to the sampled oviduct contained only follicles smaller than 5 mm in diameter, with no follicle larger than 5 mm present in the contralateral ovary and no corpus luteum was present in either ovary. The large follicle group included isthmus cells from reproductive tracts in which the ipsilateral ovary presented one or more follicles measuring 8–10 mm in diameter, with no follicle larger than 8 mm in the contralateral ovary, and absence of CL in both ovaries. The CL group comprised isthmus cells collected from oviducts in which the ovary ipsilateral contained an active corpus luteum, with no follicle larger than 5 mm present in the contralateral ovary. For inclusion in this group, the ovary was required to exhibit a corpus luteum covering at least 80% of the ovarian surface, with a coloration ranging from dark brown to orange and clear evidence of peripheral vascularization features indicative of recent luteal activity, corresponding to diestrus, as previously described by Ireland et al. (1980).

Initially, isthmus cells from the three experimental groups were cultured in vitro for 24 hours to allow the formation of cellular aggregates. At the end of this period, the number of aggregates formed in each group was quantified. For this experiment, each experimental group (SF, LF, CL) included five replicates, each composed of oviducts from two distinct animals, resulting in a total of 30 oviducts, with 10 assigned to each group. Next, an additional oviductal tissues from each group were cryopreserved in liquid nitrogen at –196 °C and subsequently subjected to gene expression analysis to evaluate *ESR1*, *ESR2*, and *PGR* profile in the isthmus. For PCR analysis, three replicates per group were performed, each consisting of two oviducts from distinct animals per replicate. resulting in a total of 18 oviducts, with 6 assigned to each group. Only samples that passed quality control were included to ensure consistency and accuracy of the results.

### Isolation and culture of isthmus epithelial cells

Oviduct-ovary complexes were collected at a local slaughterhouse and transported in 0.9% saline solution at 35 °C to the Animal Reproduction Laboratory at the Federal University of Espírito Santo, Alegre-ES.

All harvest and processing procedures followed the protocol described in Carvalho et al. (2018). To summarize, oviducts were carefully dissected from surrounding tissues, and a 6 cm segment proximal to the uterine-oviductal junction was excised. Each isthmus was placed individually in disposable 60 × 15 mm Petri dishes (ProLab, São Paulo, Brazil) and immersed in TALP medium (TCM-199 with Hanks’ salts, Gibco BRL®) supplemented with 10% fetal bovine serum (FBS, Gibco BRL®) and 100 IU/mL amikacin (Pareun, Ourofino, São Paulo, Brazil). The isthmuses were then gently compressed longitudinally between glass slides (Vision Glass, Rio Grande do Sul, Brazil), releasing epithelial cells into the TALP medium. This cell suspension was subsequently collected into 15 mL Falcon tubes (Kasvi, Paraná, Brazil) and allowed to sediment.

**Figure 1:**
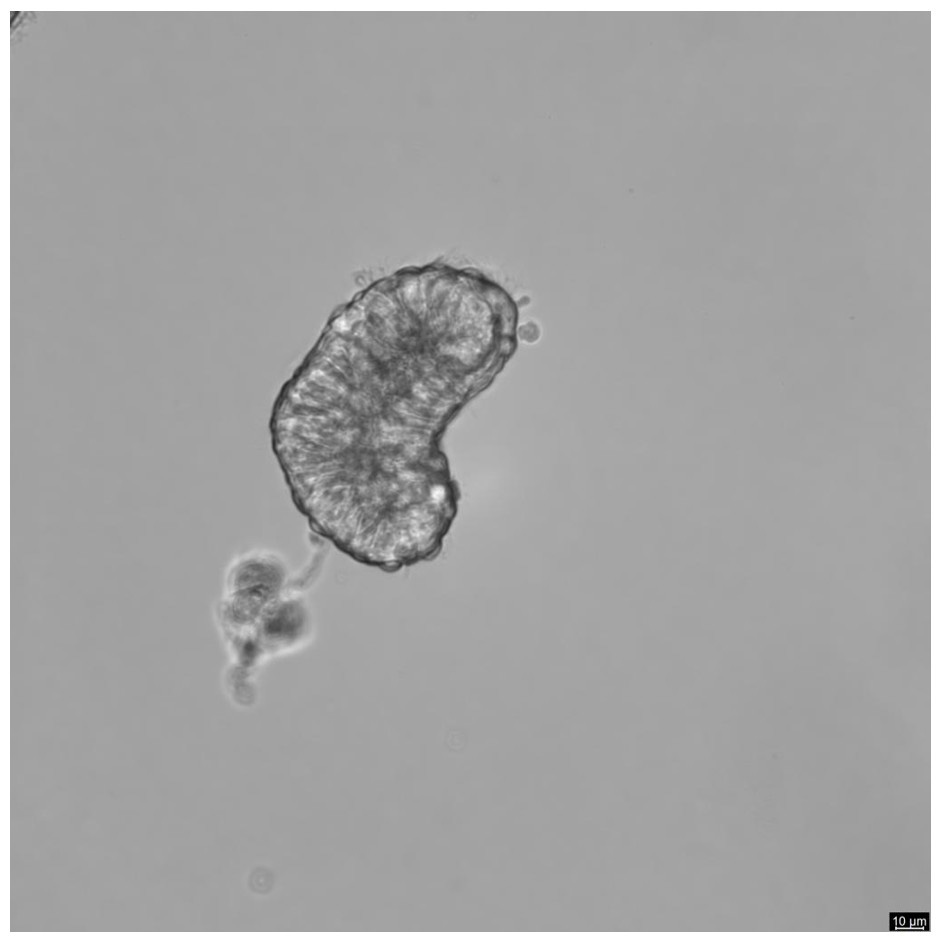
Phase contrast image (magnification ×40) showing an isthmus cellular aggregate after 24 h of in vitro culture period. Scale bar = 10 μm.

After cellular sedimentation, the supernatant was removed and 5 mL of fresh TALP medium was added to the pellet. Cells were disaggregated by sequential pipetting and transferred to disposable 50 × 15 mm Petri dishes (ProLab, São Paulo, Brazil), each containing a final volume of approximately 25 mL of TALP medium. The cultures were incubated at 39 °C with 5% CO_2_ for 24 hours to promote the formation of spherical aggregates of isthmus epithelial cells as explants (Figure 1). After 8 hours of incubation, 50% of the medium was replaced. At the end of the 24 h period, the cellular aggregates were evaluated using a stereomicroscope at 2× magnification (Nikon SMZ 645, Tokyo, Japan).

Visual counts of the absolute number of cellular aggregates formed on each Petri dish (one from each replicate of each experimental group) were performed and recorded to quantify oviductal isthmus cell explants. For this study, only spherical-shaped cellular aggregates ranging from 150 to 450 μm in perimeter were selected.

### Total RNA extraction from isthmus epithelium

Isthmus segment were homogenized in an automatic homogenizer in the presence of TRIzol Reagent (Invitrogen - Life Technologies) at a ratio of 1 mL of TRIzol for every 100 mg of tissue. After 5 minutes of incubation at room temperature (RT), 200 µl of chloroform were added for each 1 mL of macerated tissue-TRIzol suspension to separate total RNA from cellular remnants. The mixture was then incubated at RT for 3 minutes.

Tubes containing this mixture were then centrifuged at 15,700 ×g for 15 minutes at 4˚C, at which point the less dense aqueous phase was transferred to a new tube. Total RNA was precipitated from this new tube via the addition of 0.5 mL of isopropyl alcohol. This mixture was incubated for 10 minutes at RT, after which a new 15,700 ×g centrifugation at 4˚C for 10 minutes was performed. The resulting pellet was washed in 75% ethanol (prepared with water treated with 0.01% diethylpyrocarbonate - DEPC, for the removal of RNases) and resuspended in 20 µL of similarly DEPC-treated water. After resuspension, the mixture was incubated on a heating block for 10 minutes at 55 ˚C. RNA purity and concentration were assessed using a NanoDrop^®^ ND-1000 UV/Vis Spectrophotometer, samples with a 260/280 ratio of 1.9 or greater were used for analysis.and electrophoresis was performed on 1% agarose gel.

### cDNA synthesis and gene expression in isthmus epithelial cells

Approximately 1 µg of total RNA was used for complementary DNA (cDNA) synthesis with the RevertAid™ H Minus First Strand cDNA Synthesis Kit (Fermentas), following the manufacturer’s instructions. For the initial step, 0.5 µg of Oligo(dT) was added to the total RNA and incubated at 65 °C for 5 minutes. Subsequently, 2 µL of 10 mM dNTP mix, 4 µL of enzyme buffer [250 mM Tris-HCl (pH 8.3), 250 mM KCl], 20 U of RiboLock RNase Inhibitor, and 200 U of RevertAid H Minus M-MuLV Reverse Transcriptase were added to the reaction mixture. This reaction was incubated at 42 ˚C for 1 hour, followed by enzymatic denaturation for 5 minutes at 70 ˚C. The cDNA obtained was stored in a -20 ˚C freezer. Template messenger RNA sequences for prime design were obtained from *GenBank* (http://www.ncbi.nlm.nih.gov/gene, Table 1), corresponding to mRNA fragments of genes specific to bovine (*Bos taurus taurus*). Primer design for both forward and reverse oligonucleotides was carried out using the Primer3 software (http://bioinfo.ut.ee/primer3-0.4.0/), with a predicted amplicon size of approximately 200 bp. Primer specificity was assessed using BLASTN (http://blast.ncbi.nlm.nih.gov/), and primer pairs were further evaluated for secondary structures and thermodynamic properties using the NetPrimer tool (http://www.premierbiosoft.com/netprimer/). RT-qPCR was performed using the iTAqTM Universal SYBR^®^ Green Supermix (Bio-Rad). 0.4 µM of each primer and the cDNAs derived from each experimental group’s oviductal isthmuses were diluted at a 1:10 ratio. Amplification reactions of each fragment were performed using the following steps: an initial 2 minutes at 95˚C, followed by 40 cycles of 15 seconds at 95˚C, 15 seconds at 60˚C and 20 seconds at 72˚C, and finally a period of 5 minutes at 72˚C. Relative quantification was performed in technical triplicate, using *GAPDH* as the endogenous reference gene. RT-qPCR analysis (three biological replicates per group; n= 2 isthmus segment per group per replicate), was performed on a Rotor Gene 3000 Real Time PCR System, and differential expression analysis was performed using the REST 2009 software, developed by Pfaffl, (2001) and Pfaffl et al. (2002). All qPCR analysis protocols were performed in accordance with the protocol previously described (Nascimento et al., 2023).

**Table 1:** Primer sequence of the *ESR1, ESR2*, *PGR* and *GAPDH,* genes (description of genes, and primers sequences 5’—3’).

**Gene**	**Forward**	**Reverse**
*ESR1*	TCCATGGAGCACCCAGGAAAGC	CGGAGCCGAGATGACGTAGCC
*ESR2*	TCCTGAATGCTGTGACCGAC	GTGCCTGACGTGAGAAAGGA
*PGR*	CCCGCCCTATCTCAACTACC	CCCTTCCATTGCCCTTTT
*GAPDH*	ATGCCTGCTTCACCACCTT	CCAGAACATCATCCCTGCTT

*ESR1*: gene that encodes the transcription of ERα (E2-α receptor); *ESR2*: gene that encodes the transcription of ERβ (E2-β receptor); *PGR*: gene that encodes the transcription of progesterone (P4) receptor; *GAPDH*: glyceraldehyde-3-phosphate dehydrogenase.

### Statistical analysis

The number of cellular aggregates was tested for normality using the Shapiro–Wilk test and compared using the Kruskal–Wallis test followed by Dunn’s post hoc test. All analyses were performed in R version 3.6.1 (R Core Team, 2019), with the significance level set at 5%. Relative gene expression analyses were performed among the LF, SF and CL groups using the REST 2009 software, developed by Pfaffl and collaborators (2001, 2002), with graphics obtained using GraphPad Prism (version 9.0, San Diego, CA, USA).

## Results

### The presence of follicles in the ovary enhances the stimulus for isthmus cells aggregation

To investigate the effects of ovarian structures (small follicles, large follicles, and corpus luteum) on the isthmus epithelium reaggregation, cells samples of each group were cultured in vitro for 24 h. A greater number of cellular aggregates was observed in the LF (364.0 ± 139.1) group compared to the SF (197.6 ± 127.7; *p* = 0.035) and CL groups (8.0 ± 11.0; *p<* 0.001). Additionally, the number of isthmus explants was higher in the SF (*p* = 0.002) than in the CL group (Table 2). The results demonstrated that the presence of follicles in the ovarian surface had a significant impact on the formation of cellular aggregates in the isthmus. Furthermore, as follicle size increased, the aggregation potential of isthmus cells also increased.

**Table 2:** Mean number of oviductal cell aggregates formed in each experimental group.

**Number of isthmus cellular aggregates**	
Group	Mean ± SD
SF (Fol. <5 mm)	197.6 ± 127.7 ^a^
LF (Fol. 8-10mm)	364.0 ± 139.1 ^b^
CL	8.0 ± 11.0 ^c^

SF (Small Follicle Group): Reproductive tracts in which the ovary ipsilateral to the analyzed oviduct contained follicles smaller than 5 mm in diameter, with no CL present in either ovary. LF (Large Follicle Group): Reproductive tracts in which the ovary ipsilateral to the analyzed oviduct contained one or more follicles measuring 8–10 mm in diameter, with no corpus luteum (CL) present in either ovary. CL (Corpus Luteum Group): Reproductive tracts in which the ovary ipsilateral to the analyzed oviduct presented a corpus luteum. Different indicate statistically significant differences.

### Ovarian structures regulate the expression of steroid hormone receptor genes in the isthmus epithelium

**Figure 2:**
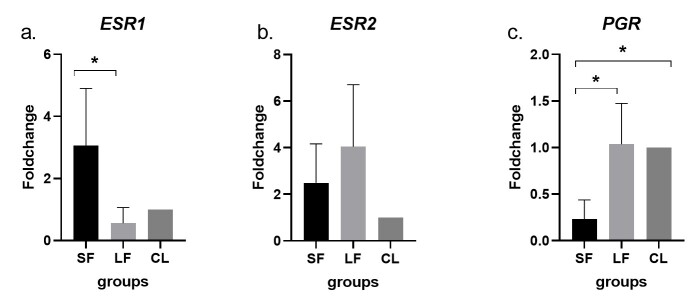
Expression of Steroid Hormone Receptor Genes in Isthmus Cells from Small (SF) and Large Follicles (LF) and Corpus Luteum (CL) group. a. ESR1 (encoding the estrogen receptor alpha, ERα); b. ESR2 (encoding the estrogen receptor beta, ERβ); c. PGR (encoding the nuclear progesterone receptor). Asterisks above the bars within the same graph indicate statistically significant differences (p < 0.05). Bars represent mean ± standard error of the mean (SEM).

To assess the influence of ovarian structures on the expression of steroid hormone receptors, gene expression analysis was conducted on isthmus cells immediately after tissue collection. The *ESR1* showed higher expression levels in cells from the SF group compared to the LF group (*p<* 0.01) (Figure 2a). In contrast, *ESR2* expression was consistent across all groups (Figure 2b). Regarding *PGR* expression, significantly higher levels were observed in the CL group compared to the SF group (*p<* 0.001). Additionally, the LF group also exhibited greater *PGR* expression than the SF group (*p<* 0.001) (Figure 2c). The results showed that variations in follicle size and the CL presence in the ovarian surface shape the expression of estrogen and progesterone receptor genes in isthmus cells.

## Discussion

From the time of ovulation, the oviduct acts as a shelter for crucial events that culminate in the early stages of embryonic development. The final stages of oocyte maturation, fertilization, and the first embryonic cleavages occur within the oviductal environment (Coy et al., 2012). These dynamic steps dictate the morphological and functional state of the oviduct segments: the infundibulum, ampulla, and isthmus. However, before ovulation, the oviductal microenvironment is already regulated by periovulatory sex steroid hormones (Bauersachs et al., 2005; Binelli et al., 1999) which are primarily secreted by ovarian structures such as dominant follicles and the corpus luteum. Nevertheless, information regarding how the structures in the ovarian surface impact the oviductal epithelium remains limited. Thus, the present work aimed to compare the behavior of isthmus epithelium collected in the presence of different traits of ovary surface (small follicles, large follicles and the corpus luteum).

This study demonstrated that isthmus cells from reproductive tracts containing different ovarian structures exhibit distinct aggregation capacities after 24 h of in vitro culture, as well as differential expression of steroid hormone receptor genes. Sex steroid hormones such as estrogen and progesterone, acts as regulators of oviductal transcriptome and function throughout estrous cycle (Gonella-Diaza et al., 2015; Hazano et al., 2021, 2019). It is well established that during ovarian follicle development, there is a gradual increase in systemic estrogen concentrations. As the follicle reaches the pre-ovulatory stage, estrogen secretion peaks (Smith et al., 1975). Following ovulation, a major shift in hormonal secretion occurs. With the formation of the corpus luteum, a progressive rise in progesterone levels begins, with the highest systemic concentrations typically observed around day 11 post-ovulation (Ireland et al., 1980). Therefore, the presence of small and large follicles, as well as an active corpus luteum, indicates that the endocrine milieu is under the influence of increasing concentrations of estrogen (secreted by follicles), followed by increasing progesterone concentrations (secreted by the corpus luteum) (Ireland et al., 1979; Smith et al., 1975).

Hormonal analyses were not performed in this study to directly verify the influence of ovarian-derived sex steroid hormones on isthmus cells. Nevertheless, the different ovarian structures associated with the distinct patterns observed in cellular aggregation and steroid hormone receptor gene expression strongly imply that the endocrine milieu underwent distinct variations in estrogen and progesterone concentrations among the experimental groups. Such hormonal variations are described to profoundly modulate both the phenotypic behavior (Abe, 1994) and transcriptional profiles of isthmus cells (Hazano et al., 2021).

In this study, isthmus cells from the LF group exhibited a greater aggregation capacity compared to those from the SF and CL groups. The formation of cellular aggregates occurs through increased intercellular connectivity and the proliferation of small cellular units in culture (Ferraz et al., 2018). It is well established that dominant and pre-ovulatory follicles secrete higher concentrations of estrogen (Smith et al., 1975). Based on this, the greater aggregate formation observed in the LF group suggest that estrogen may play an indirect stimulatory role by promoting mitogenic activity in isthmus cells. Estrogen is well recognized as a mitogenic hormone (Cerny et al., 2016). One of the mechanisms through which estrogen regulates the mitogenic activity of cells is by stimulating the production of growth factors such as IGF-1 and IGF-2 (Frasor et al., 2003), which in turn activate key signaling pathways, including MAPK, ERK1/2, and PI3K/Akt- central to cellular proliferation (Ipsa et al., 2019; Rowlinson et al., 2008; Shao et al., 2007b). Additionally, this mitogenic effect appears to be proportional to the level of estrogenic influence, which increases with follicular development. Although aggregate formation does not provide definitive evidence of mitogenesis, and further studies are needed to explore this finding, these results support the concept that the presence of follicles on the ovarian surface primes the oviductal epithelium by enhancing its proliferative capacity.

Interestingly, the CL group exhibited the lowest capacity for cellular aggregation. This finding may be associated with the predominance of progesterone in the endocrine milieu during the luteal phase (CL presence), which is known to exert antiproliferative effects on epithelial tissues and as well as suppress ciliation and secretory activity in the oviduct (Slayden et al., 2022). These physiological effects could explain the reduced ability of isthmus cells from the CL group to form aggregates. This observation supports the notion that the functional state of the oviductal epithelium undergoes adaptive changes in response to the reproductive phase.

Regarding gene expression analysis, despite lower *ESR1* expression in the LF group compared to the SF group, LF cells exhibited greater aggregate formation. This paradox may be explained by the observation that, although estrogen typically upregulates *ESR1*, high estrogen concentrations can suppress its own receptor expression (Shao et al., 2007a, b; Tibbetts et al., 1998). Thus, the lower *ESR1* levels in the LF group may, paradoxically, indicate a stronger estrogenic influence than in the SF group.

In addition, another dual effect of estrogen occurs in regulating *PGR* expression. This study demonstrated that *PGR* expression was significantly higher in the CL group compared to the SF group and elevated in the LF group relative to the SF group. It is well established that progesterone stimulation leads to the downregulation of its nuclear receptors (Hazano et al., 2021; Takahashi et al., 2016). However, Ulbrich et al. (2003) demonstrated that PR-B levels (an isoform of nuclear P4 receptor) remain consistently high throughout the luteal phase, with particularly strong expression in the isthmus region. Based on these findings, we hypothesize that high circulating progesterone concentrations may differentially modulate the expression of the isoforms (PR-A and PR-B) of the nuclear progesterone receptor. Nevertheless, further studies evaluating PR-A and PR-B separately are warranted to confirm this hypothesis.

On the other hand, estrogen is described to stimulate and/or inhibit *PGR* expression depending on its concentration and receptor interactions. At low concentrations, such as those likely present in the SF group, estrogen may induce downregulation of *PGR*, while at higher concentrations, such as in the LF group, it may stimulate *PGR* expression (Berisha et al., 2002; Hazano et al., 2021; Ulbrich et al., 2003). This concentration-dependent regulation could explain the observed gene expression patterns: lower *PGR* levels in the SF group and higher levels in the LF and CL groups. In a broader context, we postulate that both mechanisms may have acted synergistically to account for the increased *PGR* expression observed in the CL group: the estrogenic stimulus present in the SF group may have contributed to lower *PGR* expression, whereas the elevated progesterone levels in the CL group may have promoted *PGR* upregulation.

## Conclusion

This study demonstrates that the ovarian status, particularly the presence of large follicles or a corpus luteum, modulates the behavior and gene expression profile of bovine oviductal isthmus cells. Specifically, isthmus cells from reproductive tract with ovaries bearing large follicles (and no corpus luteum) exhibited greater aggregation capacity (i.e., increased cellular aggregates formation), likely influenced by estrogenic activity, while progesterone may exert an inhibitory effect. Additionally, our findings suggest that these functional changes are primarily regulated through the differential expression of *ESR1* and *PGR*. However, further studies are required to confirm whether these effects are directly mediated by the steroid hormones secreted by ovarian structures. Based on these findings, this study provides evidence that the isthmic epithelium is primed throughout the estrous cycle to support the biological events occurring in the oviductal environment.
